# Behavioral Response of *Corophium volutator* to Shorebird Predation in the Upper Bay of Fundy, Canada

**DOI:** 10.1371/journal.pone.0110633

**Published:** 2014-10-29

**Authors:** Elizabeth C. MacDonald, Elisabeth H. Frost, Stephanie M. MacNeil, Diana J. Hamilton, Myriam A. Barbeau

**Affiliations:** 1 Department of Biology, Mount Allison University, Sackville, New Brunswick, Canada; 2 Department of Biology, University of New Brunswick, Fredericton, New Brunswick, Canada; University of Western Australia, Australia

## Abstract

Predator avoidance is an important component of predator-prey relationships and can affect prey availability for foraging animals. Each summer, the burrow-dwelling amphipod *Corophium volutator* is heavily preyed upon by Semipalmated Sandpipers (*Calidris pusilla*) on mudflats in the upper Bay of Fundy, Canada. We conducted three complementary studies to determine if adult *C. volutator* exhibit predator avoidance behavior in the presence of sandpipers. In a field experiment, we monitored vertical distribution of *C. volutator* adults in bird exclosures and adjacent control plots before sandpipers arrived and during their stopover. We also made polymer resin casts of *C. volutator* burrows in the field throughout the summer. Finally, we simulated shorebird pecking in a lab experiment and observed *C. volutator* behavior in their burrows. *C. volutator* adults were generally distributed deeper in the sediment later in the summer (after sandpipers arrived). In August, this response was detectably stronger in areas exposed to bird predation than in bird exclosures. During peak predator abundance, many *C. volutator* adults were beyond the reach of feeding sandpipers (>1.5 cm deep). However, burrow depth did not change significantly throughout the summer. Detailed behavioral observations indicated that *C. volutator* spent more time at the bottom of their burrow when exposed to a simulated predator compared to controls. This observed redistribution suggests that *C. volutator* adults move deeper into their burrows as an anti-predator response to the presence of sandpipers. This work has implications for predators that feed on burrow-dwelling invertebrates in soft-sediment ecosystems, as density may not accurately estimate prey availability.

## Introduction

Predation is a key process contributing to the organization of ecological communities [1], [2], [3], [4], [5]. Predators have direct consumptive effects on prey populations while also eliciting strong indirect effects [6], [7], [8]. Prey can adopt avoidance strategies in response to predation, including biochemical, morphological and behavioral defenses [9], [10], [11], [12], [13]. Habitat structure can facilitate predator avoidance by providing refuges for prey [14], [15]. Even in structurally simple environments, such as soft-sediment ecosystems, prey can take advantage of three-dimensional structure [16]. Soft-sediment invertebrates may burrow into the substratum [17], [18], [19], or create tubes to evade predators [20], [21].

The amphipod *Corophium volutator* is abundant on intertidal mudflats in the upper Bay of Fundy, Canada [22], [23]. *C. volutator* construct “U”–shaped burrows, which reduce predation risk [24] and dehydration [25], [26]. Based on cursory observation, burrows typically occur in the upper 5 cm of sediment [27], [28], [29] but may extend 1–10 cm into the mud [30]. Most individuals remain burrowed as the tide recedes [29], [31]; however, some will crawl on the surface for a short time after exposure [32], [33], [34].

*C. volutator* are vulnerable to predation by benthic fish, polychaetes, and other intertidal invertebrates [35], [36], [37], as well as disturbances and predation by mud snails (*Nassarius obsoletus* [*Ilyanassa obsoleta*]) [38], [39]. Their greatest predation risk is concentrated during the late summer when hundreds of thousands of migrating Semipalmated Sandpipers (*Calidris pusilla*) stopover on mudflats in the upper Bay of Fundy [40]. Individual birds stay for 2–4 weeks [40], [41], feeding intensively on *C. volutator*, as well as other invertebrates and biofilm [42]. Birds can consume thousands of amphipods per day [43], and typically double their mass during their short stay in this region [43], [44], [45].

Crawling *C. volutator* are highly vulnerable to predation and sharp declines in crawling behavior coincide with the arrival of sandpipers [24], [46]. Boates et al. [46] found that surface-crawling activity by adult males fell by 97% after sandpipers arrived, but was concurrently unchanged at another mudflat that birds did not visit. They also noted, over several years, that cessation of crawling coincided directly with arrival of birds, and could not be fully explained by loss of individuals through predation. In Europe, foraging Common Redshanks (*Tringa totanus*) caused the number of crawling *C. volutator* individuals in close proximity to decline significantly, suggesting *C. volutator* may detect, and respond to, changes in substratum pressure [47]. Similarly, Boates et al. [46] observed that fewer individuals crawled on sediment visited by sandpipers than on adjacent areas where birds had not walked. This suggests that *C. volutator* responded directly to sandpipers rather than by using general seasonal cues.

Sandpipers can probe into burrows to obtain prey; however, their success is limited by bill length [48], [49], which, in New Brunswick, averages 21.5 mm for females and 19.6 mm for males [50]. Thus, *C. volutator* may be able to escape predation by burrowing beyond the reach of foraging birds [51]. This avoidance mechanism, sometimes termed prey depression [52], temporarily reduces available prey [47], [52], [53], and has been inferred from declines in prey crawling activity [47], [53] and reduced feeding rates for redshanks in areas recently occupied by conspecifics [54], [55]. It has also been speculated to influence shorebird behavior on Bay of Fundy mudflats [56], [57]. Coulthard and Hamilton [38] found proportionally fewer *C. volutator* adults near the sediment surface in the presence of high densities of mud snails at one mudflat, further suggesting that *C. volutator* have some capacity to adjust their position in the sediment in response to disturbances and/or predation.

We conducted three complementary studies examining behavioral responses of *C. volutator* to Semipalmated Sandpiper predation on mudflats in the Bay of Fundy. First, we designed a field experiment to test if the presence of shorebird predators affects the vertical distribution of *C. volutator* adults. We quantified their distribution in the sediment before and during the period when shorebirds were present, in plots where birds were either present or excluded. Next, we monitored *C. volutator* burrow depth by making polymer casts in the field before, during and after the period of peak shorebird abundance. Finally, we conducted a lab experiment where we simulated sandpiper pecking to test if *C. volutator* females moved deeper in their burrows in response to perceived predation risk. We expected that *C. volutator* would exhibit signs of predator avoidance in the presence of shorebirds. Specifically, we predicted they would be lower in the sediment when exposed to shorebird predation or perceived predation. This could occur through construction of deeper burrows and/or increasing time spent lower in their burrows. In summary, this study allowed us to examine the effect of shorebird predators on *C. volutator* vertical distribution in the sediment, and to determine if adult *C. volutator* exhibit predator avoidance behavior in the presence of foraging sandpipers.

## Materials and Methods

### Experiment 1: Predator exclusion in the field

**Figure 1:**
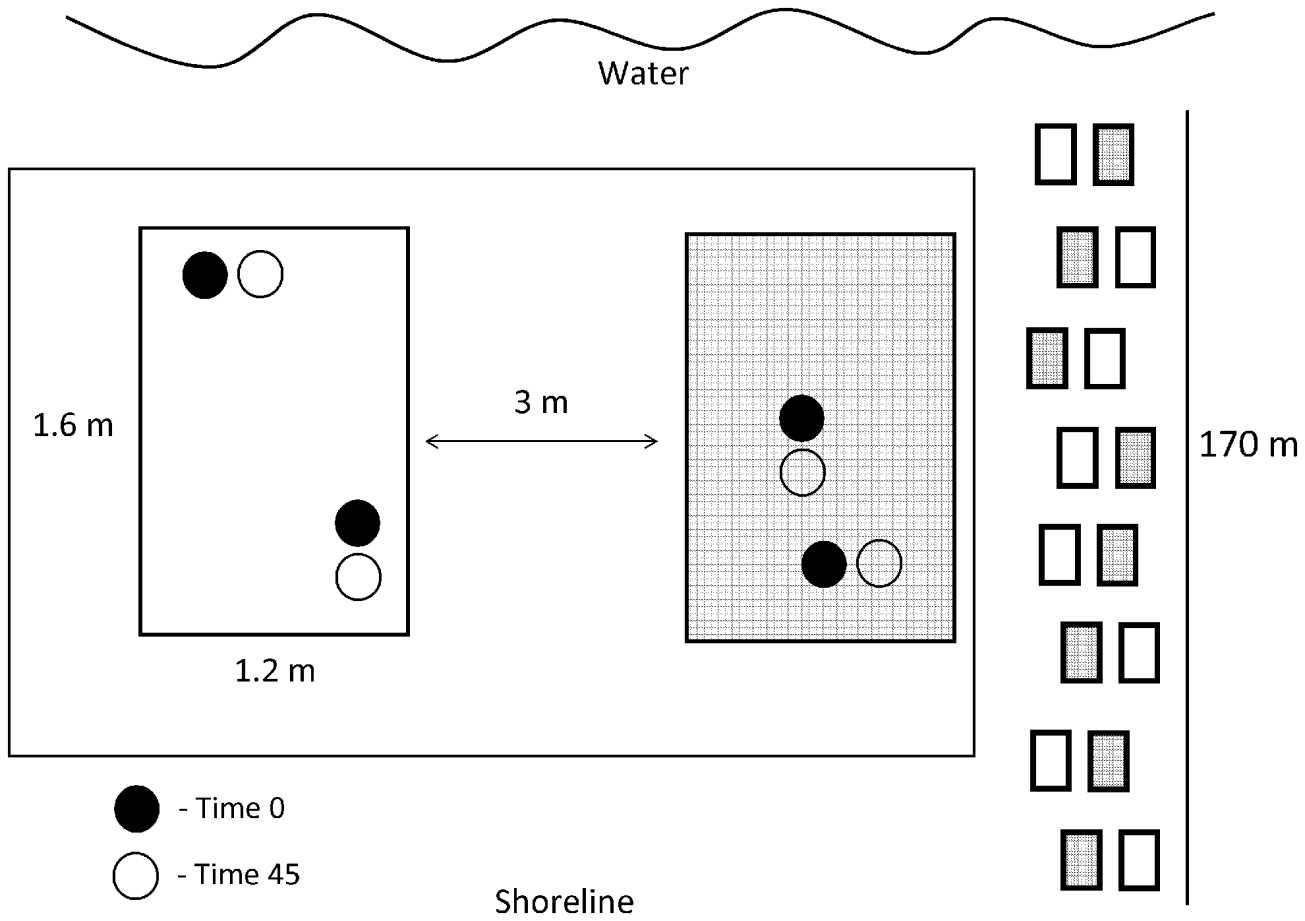
Design of the predator exclusion experiment with spatial blocks at Pecks Cove in summer 2007 (not to scale). Each pair of small rectangles represents a spatial block. Blocks were set up between 130 and 300 m from shore. Each block consisted of two treatment levels: a netted bird exclosure (with hatching) and a control plot (without hatching). Two replicate samples were taken within each treatment plot immediately following the ebb tide (time 0), and a second pair of samples were taken ∼5 cm from the first 45 min later. Locations from which samples were taken within each plot were randomly selected in advance.

We conducted a predator exclusion experiment, using a block design, to compare vertical distribution of *C. volutator* adults in areas with and without shorebird predators. We set up the experiment on 8 July 2007 at Pecks Cove in the Bay of Fundy, New Brunswick, Canada (45°45'N, W 64°28'W). Pecks Cove has an intertidal mudflat extending ∼850 m from shore and supports migrating sandpipers each summer. Pecks Cove is not privately owned or protected, thus we did not require specific permission to sample at this site. We established 8 spatial blocks, each consisting of two treatment levels: a netted exclosure preventing shorebird predation and a control plot allowing free access to shorebirds (Figure 1). These treatment plots (caged and uncaged) were 1.6 m×1.2 m and were spaced 3 m apart within a block. These dimensions were chosen as they allowed us to sample an entire plot without disrupting the sediment within it, and previous work indicated that cages of this size were appropriate for detecting predator effects [58]. Exclosures consisted of bamboo stakes with a top made of clear plastic mesh (12.5-mm openings). The stakes were pounded 0.3 m into the mud, leaving the top of the exclosure 15 cm above the sediment. The sides of the exclosures were open, which avoided disruption of water flow [37]. Flagging tape streamers were tied to the top frame of the exclosure to help deter birds. Additional cage controls, typically consisting of mesh coverage of the top and some sides [59], were not possible or necessary because the exclosures already had open sides. We did not expect shading from the mesh top to be problematic as similar cages constructed with a finer mesh generated minimal reductions in photon flux density [37]. We marked control plots with bamboo stakes.

We regularly estimated shorebird density in a 400-m radius of the experiment within 2-h of a daytime low tide starting on 1 July, and increased the frequency of these bird counts to at least 3 times/week starting on 25 July 2007 (when shorebirds started to arrive at Pecks Cove). Additionally, beginning on 9 August, we estimated percent shorebird footprint cover in exclosures and control plots as an index of habitat use [60] ∼4 times/week at low tide.

From 10 July to 19 August, we collected samples from exclosures and control plots at ∼10-d intervals, totaling 5 sampling dates. In each plot, we counted crawling *C. volutator* individuals in a 0.25 m×0.25 m quadrat and recorded sediment temperatures at the surface and 5 cm depth (corresponding to the top and bottom of our sampling device) with a digital thermometer. We then collected sediment cores using a stratified sampling device divided into four horizontal layers (described in Coulthard and Hamilton [38]): 0–0.5, 0.5–1.5, 1.5–3.0, and 3.0–5.0 cm from the sediment surface. On each sampling date, we collected two replicate cores from exclosures and control plots in each spatial block immediately after mud exposure (time 0) and again 45 min later (Figure 1). Samples were not collected from a 10-cm buffer around the edge of plots, and different portions of the plot were sampled on each subsequent round. It is possible that individuals moved vertically within their burrows in response to sampling. However, the process was quick (<30 s to collect a sample and section the layers) and the disruptive nature of sampling was consistent among treatment levels and sampling dates, so any observed differences should be independent of effects due to sampling method or researcher presence.

We rinsed each layer of a core separately through a 250-µm sieve [61], and preserved the contents in 95% ethanol. We measured all *C. volutator* individuals (body length from rostrum to telson) in each layer, and grouped the adults (>4 mm body length) into two categories: 4–6 and >6 mm. Individuals <4 mm are considered juvenile and not preferred shorebird prey (data presented in Wallace [62]), while 4–6 mm amphipods are completing sexual development but are potential prey, and the fully developed >6 mm adults are considered preferred shorebird prey [22], [37]. This protocol was developed in accordance with the policies of the Canadian Council on Animal Care and was approved by the Mount Allison Animal Care Committee (Permit Number: 07–12). We did not sample any protected species.

### Field study: Burrow casts

We measured *C. volutator* burrow depths by making polymer resin casts of areas with *C. volutator* burrows on the Pecks Cove mudflat in summer 2011, following a protocol adapted from Gingras et al. [63]. We randomly selected 3 locations (between 100 and 300 m from shore, separated by at least 100 m) three times: 25 July, 19 August and 30 August, corresponding with shorebird arrival, and near peak abundance and departure, respectively. We attempted two casts per location per time; however, not all casts were successful. We have data from three casts (one from each location) on 25 July, five casts on 19 August and all six casts on 30 August. We made casts using polyester boat-repair resin catalyzed by 8% methylethyl ketone peroxide (MEKP). Before adding the catalyst on location at low tide, we diluted the resin by 50% with acetone to decrease viscosity, enabling the resin mix to penetrate small burrow openings. Immediately after adding the catalyst, we poured the resin mix into a circular plastic form (18 cm diameter) partly pushed into the mud, very slowly so that it seeped into burrows without trapping air and water. We collected the form and hardened cast 12–18 h later at low tide.

On each cast, we sampled burrows using a stratified random design, by first categorizing all *C. volutator* burrows in three burrow width classes (i.e., strata): small (≤1 mm diameter), medium (>1 to ≤2 mm), and large (>2 mm). We attempted to measure 10 randomly selected burrows per width class per cast; however, on 40% of the width class-cast combinations, there were fewer than 10 burrows available (overall average ±SD: 8.1±2.9 burrows per width class per cast). For each selected burrow, we measured burrow depth and width (in mm).

### Experiment 2: Predator simulation in the laboratory

We conducted a laboratory experiment in summer 2011 to examine behavior of *C. volutator* females in the presence and absence of simulated shorebird pecking. We chose to use only females (≥6 mm body size) because females (i) dominate adult densities in the field [23], [64], (ii) are the main burrow-making adults [33], [65], and (iii) previous work found no difference in burrow behavior of females when alone versus when with males, but male behavior varied in the presence of females (S.M. MacNeil and M.A. Barbeau, unpubl. data). For our experiment, we constructed narrow cages using a cedar wood frame (30 cm long×30 cm high) with grooves to hold two 63-mm thick sheets of glass 0.3 cm apart (Figure S1). We filled the cages with 20 cm of mud collected from Pecks Cove and sieved through a 125-µm mesh to remove existing animals. We added nine *C. volutator* females to each cage (collected from Pecks Cove <24 h prior to set up), creating a density of 10,000 individuals/m^2^ (similar to natural conditions [23]). We conducted the experiment in three temporal blocks (15–18 August, 23–26 August and 2–5 September), each consisting of four cages, with two replicate cages per treatment level (pecking and non-pecking). All cages experienced a simulated tidal cycle (emulating the 24-h natural cycle) divided into four observation stages: immediately high (first 20 minutes following submersion), high, immediately low (first 20 minutes following emersion), and low tide. A cage had a draining hole covered with 250-µm mesh at the level of the mud surface, and tides were created by lowering or elevating the rack of cages in a large salt water tank (recirculated salt water, kept at 13.8±0.9°C and 32.5±1.2 PSU, average ±SD). The light:dark regime throughout the experiment was 15:9 h, with light levels at 1595 ±342 lux during the day.

We designed the pecking treatment to mimic natural shorebird predation behavior. Pecking took place only during the first five minutes of immediately low tide at a rate of one peck per second [57] with a probe (2-mm diameter) inserted into the mud to a maximum depth of 5 mm. We did not simulate pecking at other times during low tide, because the area in a cage is small (9 cm^2^), and the likelihood is almost zero that such a small area of mudflat would be visited more than once during a tidal cycle. Our pecking treatment had no apparent confounding behavioral effects on *C. volutator* individuals (Figures S2.1, S2.2).

We observed each cage for four 20-min observation periods (1 observation period per tidal stage) per day over a 4-d trial (for a total of 80 minutes per tidal stage per cage per trial). Each day, we randomly selected the order in which the cages were observed. For an observation period for a cage, a burrow that was fully visible and that contained a *C. volutator* individual was randomly selected as the focal burrow with the focal amphipod. We recorded the proportion of time that the focal amphipod spent at the bottom of its burrow, defined as a part of its body touching the bottom of the “U”. Other burrow behaviors were also recorded (Figures S2.1, S2.2). Animal care approval was not required at the University of New Brunswick as the experiment only involved invertebrates.

### Statistical analyses

We conducted statistical analyses in SPSS version 15.0 [66] and R version 2.10.1 [67]. We applied appropriate transformations when parametric test assumptions were violated. We evaluated main effects at p = 0.05 and investigated interactions at p≤0.10 [58]. Means are reported as ±1SE, unless otherwise indicated.

We evaluated vertical distribution of *C. volutator* (density of individuals per sediment layer) in the predator exclusion experiment in the field for each size class separately using linear mixed effects models (repeated measures design). Sediment layer (4 levels), exposure time (2 levels: 0 and 45 min after tidal recession), treatment (2 levels: presence or exclusion of shorebirds) and date (5 levels) were fixed factors, and spatial block was a random factor. We used an autoregressive (AR1) covariance structure with homogenous variances [68] and a restricted maximum likelihood method of parameter estimation. Denominator degrees of freedom are calculated using the Satterthwaite method [69], producing an approximate but unbiased F-test. To avoid pseudoreplication, we averaged the two core samples within a treatment and exposure time. When there were significant interactions with date, we conducted separate analyses for each date [70]. For all other significant interactions, we either split by one of the interacting factors or compared all combinations of treatments [68], [71] using posthoc testing with Bonferroni-adjusted p-values. For each size class, we also assessed the effect of date and shorebird treatment on overall *C. volutator* abundance (i.e., pooling over sediment layers) using linear mixed effects models.

Density of crawling *C. volutator* was also analyzed using linear mixed models with an AR1 covariance structure (repeated measures design, as above). To control for possible effects of sediment temperature on crawling behavior, we ran two additional models, one with surface temperature as a covariate and the other with temperature at 5 cm depth as a covariate. We used AIC model selection [72] to determine which of these models best predicted crawling behavior.

We compared burrow depths of *C. volutator* in the field, measured from casts, over three sampling rounds (before, during and near the end of the shorebirds' staging period). First, burrow depth was standardized for *C. volutator* body size by performing a linear regression of burrow depth versus burrow width (a proxy for *C. volutator* size). We then used the residuals from this regression in an ANOVA where sampling round (3 levels) was a fixed factor, location (3 levels) a random factor nested in round, and individual burrows were the error term. We pooled casts within a location, when both were successful, so that the unit of replication to test for the effect of round was location.

For the lab experiment, we analyzed the proportion of time spent by *C. volutator* females on the bottom of their burrow in the presence and absence of simulated pecking using a linear mixed model (repeated measures design). Treatment (2 levels: pecking, no pecking) and tidal stage (4 levels) were fixed factors, and tank was a random factor (split by tidal stage). We pooled data over the three temporal blocks, since the effect of block or interactions with block were highly non-significant (p>0.20 [73]). We applied an arcsine square-root transformation prior to analysis [71].

## Results

### Patterns in sandpiper abundance and foraging activity

**Figure 2:**
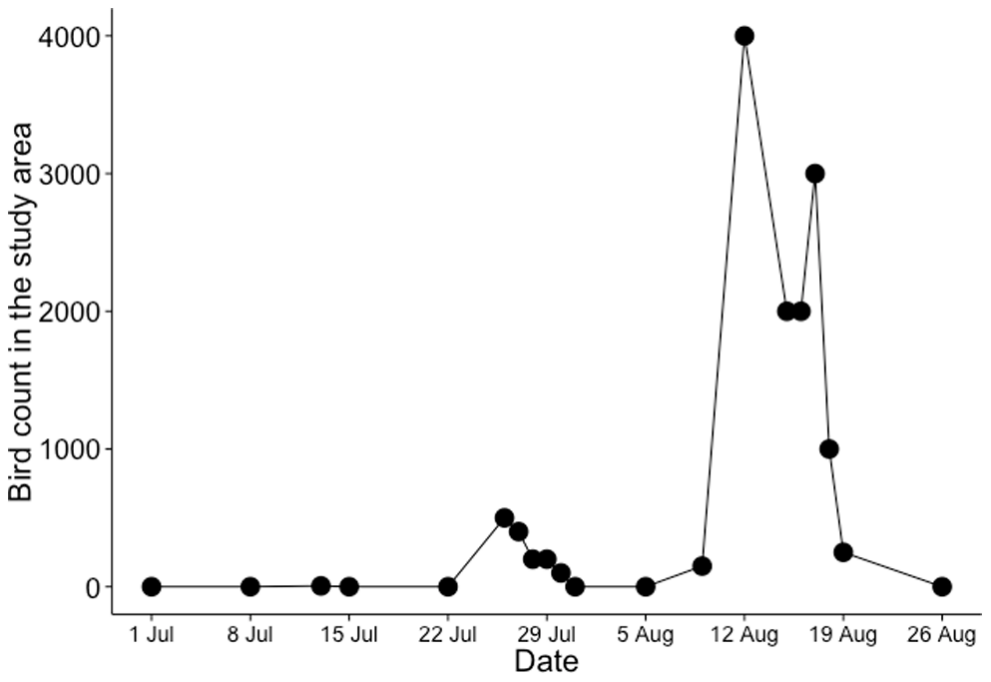
Estimates of Semipalmated Sandpiper density at Pecks Cove in 2007. Estimates of number of Semipalmated Sandpipers present within a 400 m radius of the blocked predator exclusion experiment. Estimates were taken approximately once per week until birds arrived (25 July) at which point density was estimated approximately 3 times/week for the remainder of the study.

Semipalmated Sandpipers began to arrive on 25 July in 2007, but foraging flocks were not present until early August. Abundance peaked at ∼4,000 individuals on 12 August (Figure 2) and shorebirds remained in the study area until the end of the experiment.

From 9 to 26 August, shorebird footprint cover within exclosures was very low (mean cover  = 4.2±1.1%, n = 72) and was limited to the periphery, indicating that shorebird predators were effectively excluded. As well during this time, shorebird footprint cover in control plots, which were accessible to birds, was 43.6±4.1% (n = 72).

### Seasonal trends in *C. volutator* abundance and vertical distribution

**Figure 3:**
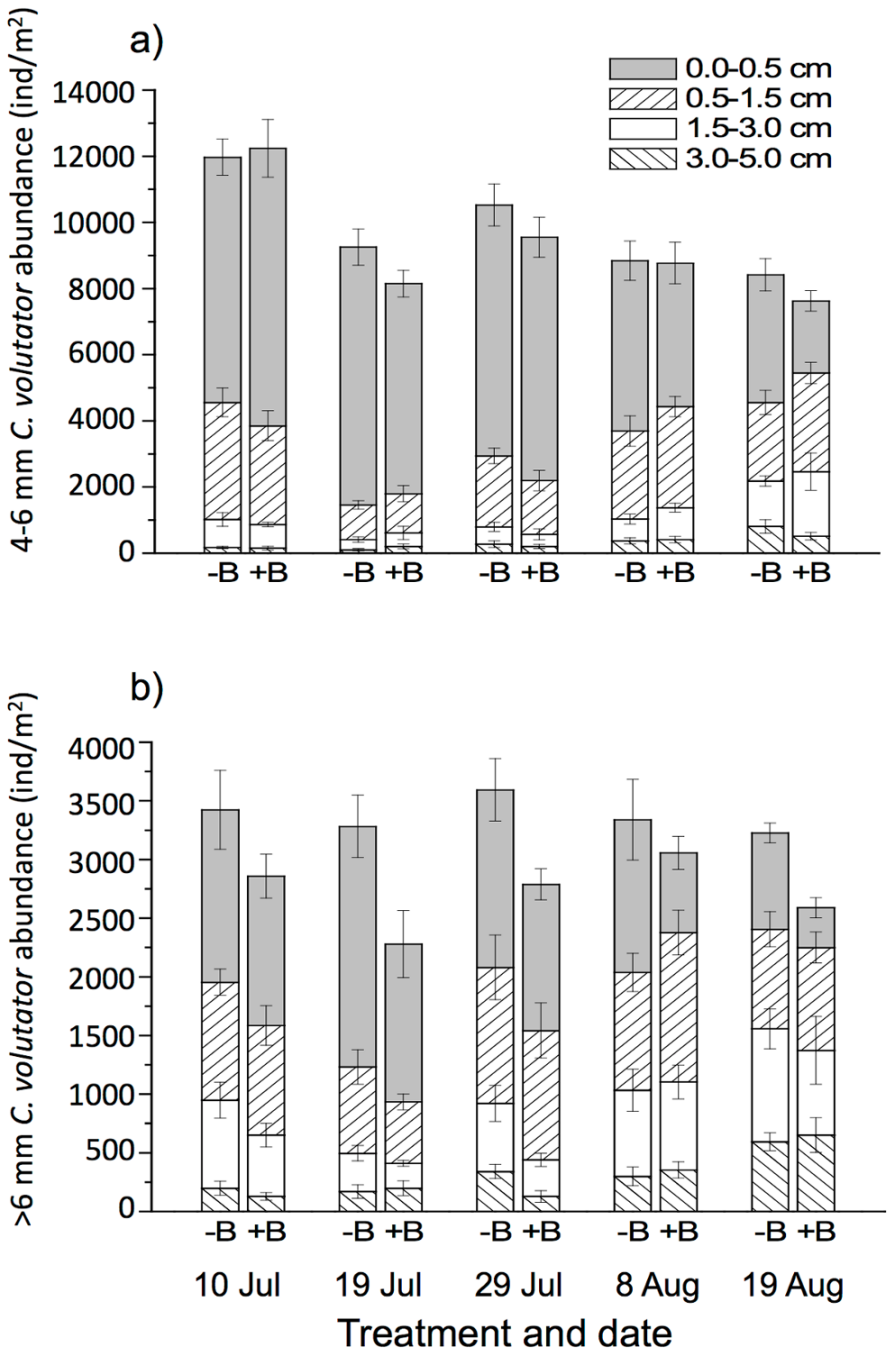
Abundance and vertical distribution of *Corophium volutator* adults sampled from the predator exclusion experiment at Pecks Cove in 2007. Abundance (individuals/m^2^) of *C. volutator* sized A) 4–6 mm and B) >6 mm in bird exclosures (−B) and control plots (+B) immediately following sediment exposure (0 min after tidal recession). Each stack represents a layer of sediment; values are mean ±1 SE (n = 8) for each combination of layer, treatment and date. Note that scale of the y-axes changes between size classes.

Overall abundance of small *C. volutator* adults (4–6 mm) varied with date (F_4,133_ = 25.7, p<0.0001; interactions with date were non-significant, p>0.59), Specifically, abundance declined between 10 and 19 July, moderately increased by 29 July, and subsequently declined slightly over the last three sampling dates (Figure 3A). The effect of date on overall abundance of large *C. volutator* adults (>6 mm) approached significance (F_4,133_ = 2.28, p = 0.06; interactions with date were non-significant, p>0.18), but no clear temporal trend could be detected (Figure 3B). In general, the population of both size classes remained high throughout the experiment (Figure 3).

We could not interpret main effects or some simple interactions from global models examining vertical distribution of *C. volutator*, because differences among layers varied with other factors (Table 1). Consequently, we conducted separate analyses for each sampling date (Table 2). This prevented statistical comparison among dates, but allowed us to examine treatment, layer and exposure effects (presented below) without elevating the risk of a type I error [70]. Visual examination of Figures 3 and S3 indicate that *C. volutator* adults tended to be deeper in the sediment layer in the summer, following arrival of shorebird predators, both in control plots where shorebirds were able to forage and in predator exclosures.

**Table 1:** Significant (α = 0.1) and approaching significant interactions from global models assessing effects of sampling date, layer (vertical position in the sediment), length of exposure (time after tidal recession) and treatment (shorebird present or excluded) on abundance (individuals/m^2^; square root transformed) of *Corophium volutator* adults sized 4-6 mm and >6 mm in an experiment at Pecks Cove in 2007.

C. volutator size	Interaction	df	F	p
4–6 mm	Treatment x date x exposure x layer	12, 428.0	1.67	0.07
>6 mm	Treatment x date	4, 217.6	1.80	0.13
	Treatment x layer	3, 461.2	2.57	0.05
	Date x exposure	4, 288.9	2.28	0.06
	Date x layer	12, 429.9	17.8	<0.0001

Results are from AR1 linear mixed models. The numerator and denominator degrees of freedom (df) are presented. For 4–6 mm *C. volutator*, main effects and simple interactions could not be interpreted due to a significant four-way interaction. For >6 mm *C. volutator*, numerous two-way interactions among all four factors made interpretation difficult. Higher-order interactions not presented were all non-significant (p>0.24).

**Table 2:** Effects of layer (vertical position in the sediment), length of exposure (time after tidal recession; Expos) and treatment (shorebird present or excluded; Treat) on abundance (individuals/m^2^; square root transformed) of *Corophium volutator* adults sized 4–6 mm and >6 mm in an experiment at Pecks Cove in 2007.

		10 Jul			19 Jul			29 Jul			8 Aug			19 Aug		
C. volutator size	Source of variation	df	F	p	df	F	p	df	F	p	df	F	p	df	F	p
4–6 mm	Layer	3, 81.1	397.7	**<0.0001**	3, 81.6	380.5	<0.0001	3, 81.3	334.7	**<0.0001**	3, 80.4	167.2	<0.0001	3, 79.9	72.6	<0.0001
	Exposure	1, 59.8	1.5	0.22	1, 69.9	3.3	0.072	1, 64.4	0.5	0.49	1, 69.5	1.5	0.22	1, 48.9	1.8	0.19
	Treat	1, 42.4	0.1	0.75	1, 49.5	0.2	0.66	1, 46.5	2.3	0.14	1, 51.1	1.0	0.31	1, 29.7	2.69	0.12
	Expos x Layer	3, 82.1	0.4	0.72	3, 84.3	5.2	**0.003**	1, 82.3	0.3	0.81	3, 81.9	0.4	0.74	3, 77.4	0.4	0.78
	Treat x Layer	3, 82.5	0.4	0.76	3, 83.3	0.7	0.54	3, 82.4	0.8	0.51	3, 81.8	4.5	**0.006**	3, 82.4	3.9	**0.011**
	Treat x Expos	1, 50.4	0.1	0.73	1, 59.0	0.01	0.93	1, 55.0	0.6	0.45	1, 60.1	0.01	0.93	1, 37.4	0.2	0.66
>6 mm	Layer	3, 79.7	63.8	**<0.0001**	3, 81.8	93.8	**<0.0001**	3, 79.3	55.7	**<0.0001**	3, 78.4	24.5	<0.0001	3, 84.4	13.3	<0.0001
	Exposure	1, 59.6	0.2	0.68	1, 66.3	8.8	**0.004**	1, 52.6	0.3	0.61	1, 55.5	0.7	0.40	1, 56.9	1.9	0.17
	Treat	1, 42.0	0.3	0.59	1, 45.9	4.9	**0.032**	1, 35.4	2.3	0.14	1, 38.1	0.01	0.94	1, 39.6	13.2	**0.001**
	Expos x Layer	3, 81.0	0.6	0.63	3, 84.4	0.7	0.57	3, 79.4	1.1	0.34	3, 79.6	0.1	0.96	3, 84.8	3.6	**0.017**
	Treat x Layer	3, 81.1	0.6	0.60	3, 84.2	1.4	0.26	3, 81.2	0.7	0.55	3, 80.3	2.4	**0.078**	3, 86.2	2.0	0.13
	Treat x Expos	1,50.1	1.2	0.28	1, 55.1	0.1	0.73	1, 43.0	2.0	0.16	1, 45.9	0.01	0.96	1, 47.3	0.2	0.69

Results are from an AR1 linear mixed model. The numerator and denominator degrees of freedom (df) are presented. When interaction terms were significant, main effects were not interpreted. Significant main effects (i.e. not part of an interaction) are in bold.

### Treatment and exposure effects on *C. volutator* abundance and vertical distribution

Overall abundance (all layers combined) varied with treatment level for both small (F_1,133_ = 3.87, p = 0.05) and large (F_1,133_ = 21.8, p<0.0001) *C. volutator* adults. In both size classes, abundance was lower in control plots relative to bird exclosures (Figure 3). There were slightly more small *C. volutator* adults in samples taken after 45 min of tidal exposure than samples taken immediately following exposure (F_1,133_ = 4.74, p = 0.03), while abundance of large *C. volutator* adults did not vary significantly with tidal exposure (F_1,133_ = 4.74, p = 0.12). All interactions were non-significant (p>0.18).

We observed a shorebird treatment effect for 4–6 mm *C. volutator* on both August sampling dates (Figures 3A, S3A), when shorebirds were present (Figure 2). Specifically, abundances among layers varied with treatment (treatment x layer interaction, Table 2). On 8 August, there was a pronounced vertical pattern in bird exclosures, with significantly more 4–6 mm amphipods in layer 1 than 2 (post-hoc testing, p<0.0001), more in layer 2 than 3 (p<0.0001), but no detectable difference between layers 3 and 4 (p = 0.41); conversely, this vertical separation was less pronounced in control plots (indicating movement downwards), with layers 1 and 2 being similar, although layer 2 still contained more amphipods than layer 3 (p<0.0001) and layer 3 more than layer 4 (p = 0.041). We observed similar vertical profiles on 19 August: most amphipods were in the top layer (layer 1> layer 2, p = 0.015) and numbers decreased with depth (layer 2>layer 3, p = 0.009; layer 3> layer 4, p = 0.004) in bird exclosures, whereas abundance did not vary significantly between the top two layers, but layer 2 had more amphipods than 3 (p = 0.035) and layer 3 had more than 4 (p<0.0001) in control plots.

For >6 mm *C. volutator*, the treatment effect was not as consistent as for small adults; during the period when shorebirds were present and actively foraging (Figure 2) the treatment x layer interaction was only detected on 8 August, and not on 19 August (Table 2). Within bird exclosures on 8 August, abundances were similar among the top three layers, with the fourth layer containing significantly fewer individuals than the first (p<0.0001, Figure 3B). In controls, amphipods were deeper in the sediment (Figure 3B, S3B); layer 2 had the most amphipods and significantly more than layer 4 (p<0.0001), but other layers did not differ significantly from one another.

A main effect of treatment (with non-significant treatment x layer interaction) was significant for large *C. volutator* adults on 19 July (just before shorebirds arrived on the mudflat) and on 19 August (when shorebirds were present), reflecting that there were generally fewer amphipods in all layers in control plots than in exclosures (Table 2). The 19 July result could not have been due to shorebirds, and did not persist to the next sampling date. On 19 August, although a treatment x layer interaction was not detected statistically, there were relatively fewer amphipods in the top-most layer compared to other layers in the presence of shorebirds than in their absence (Figure 3B, S3B).

We also observed an exposure x layer interaction for small *C. volutator* adults on 19 July and for large adults on 19 August, indicating that differences among layers varied with tidal exposure (Table 2). On 19 July, however, post-hoc testing revealed that vertical profiles at 0 and 45 min exposure were similar with more small adults in layer 1 than 2 (p<0.0001) and layer 2 than 3 (p<0.0001), while layers 3 and 4 did not differ significantly (p>0.13). On 19 August, abundance of large adults did not differ significantly among layers immediately following exposure (time 0, all comparisons between layers, p>0.05). However, 45 min later, amphipods tended to concentrate in middle layers with significantly more residing in the second and third layer than the first layer or the fourth layer (p≤0.01 for these comparisons).

### Crawling activity of *C. volutator* and temperature

**Figure 4:**
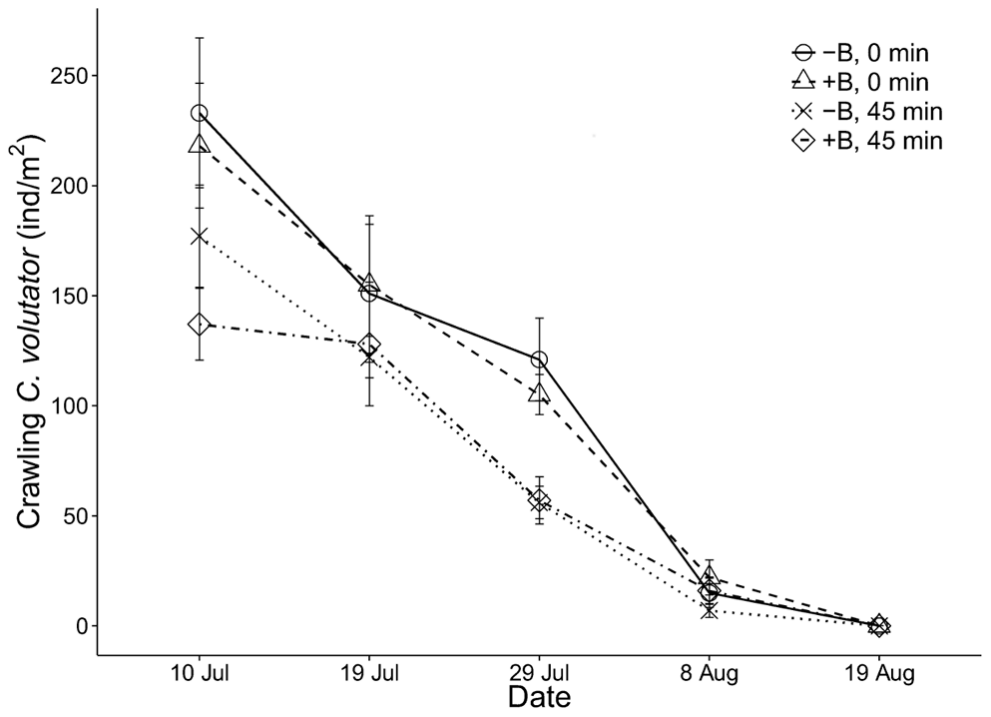
Abundance of *Corophium volutator* individuals crawling on the sediment surface throughout a predator exclusion experiment at Pecks Cove in 2007. Each symbol represents a different combination of treatment level (bird exclosures [−B] and control plots [+B]) and tidal exposure (0 min and 45 min). Values are presented as mean ±1 SE (n = 8).

Crawling declined over the summer (Figure 4), despite persistence of an adult *C. volutator* population (Figure 3). This effect varied with time since exposure (date x exposure interaction, F_4,137_ = 3.39, p = 0.01). Crawling activity declined significantly over the 45-min period (F_1,21_ = 10.0, p = 0.005) on 10 July, but there was no difference (F_1,21_ = 0.81, p = 0.38) on July 19. On 29 July (when the birds have recently arrived), crawling again declined in the 45 min after tidal recession (F_1,21_ = 29.8, p<0.0001). Due to the almost absence of crawling amphipods later in the summer (Figure 4), we did not further examine the final two sampling dates. Crawling activity did not differ significantly between treatment levels (F_1,137_ = 0.04, p = 0.85, Figure 4). Changes in crawling activity were independent of sediment temperature, since the model that best predicted *C. volutator* crawling did not include a temperature variable (Table 3). Sediment temperatures were highest during late July and early August (Table 4), when crawling activity was intermediate (Figure 4).

**Table 3:** AIC model selection results for models predicting *Corophium volutator* crawling activity during an experiment in Pecks Cove in summer 2007.

Model	AIC	ΔAIC	*w_i_*
date + treatment + exposure + date*treatment + date*exposure + exposure*treatment	189.10	0.00	0.87
date + treatment + exposure + date*treatment + date*exposure + exposure*treatment + surface temp	195.59	6.49	0.03
date + treatment + exposure + date*treatment + date*exposure + exposure*treatment +5 cm temp	193.55	4.45	0.09

Number of crawling individuals (per 0.25×0.25 m quadrat) was log transformed to improve normality and an AR1 covariance structure was used to accommodate the repeated measures sampling design. Models included the fixed effects given below and block as a random effect. “Surface temp” is the sediment temperature (°C) on the surface of the mud and “5 cm temp” is the sediment temperature at 5 cm depth. ΔAIC is the difference in Akaike's information criterion from the top model and *w*_i_ is the AIC weight, which represents the likelihood that a particular model is the best of the options available.

**Table 4:** Mean (±1 SE; n = 16, per combination of date and depth) temperature (°C) at surface and 5.0 cm sediment depths during a predator exclusion experiment at Pecks Cove in 2007.

Location	Tidal Exposure	8 July	19 July	29 July	8 Aug	19 Aug
Surface	0 min	17.7±0.2	18.3±0.05	22.5±0.2	23.4±0.2	18.8±0.1
	45 min	19.3±0.4	18.8±0.05	25.9±0.1	25.4±0.1	20.4±0.2
5 cm depth	0 min	16.3±0.05	17.9±0.03	19.7±0.05	20.0±0.08	18.8±0.1
	45 min	17.5±0.08	18.1±0.03	21.4±0.08	22.0±0.08	19.2±0.1

Measures were taken immediately following receding tide (0 min) and 45 min later.

### Burrow casts

**Figure 5:**
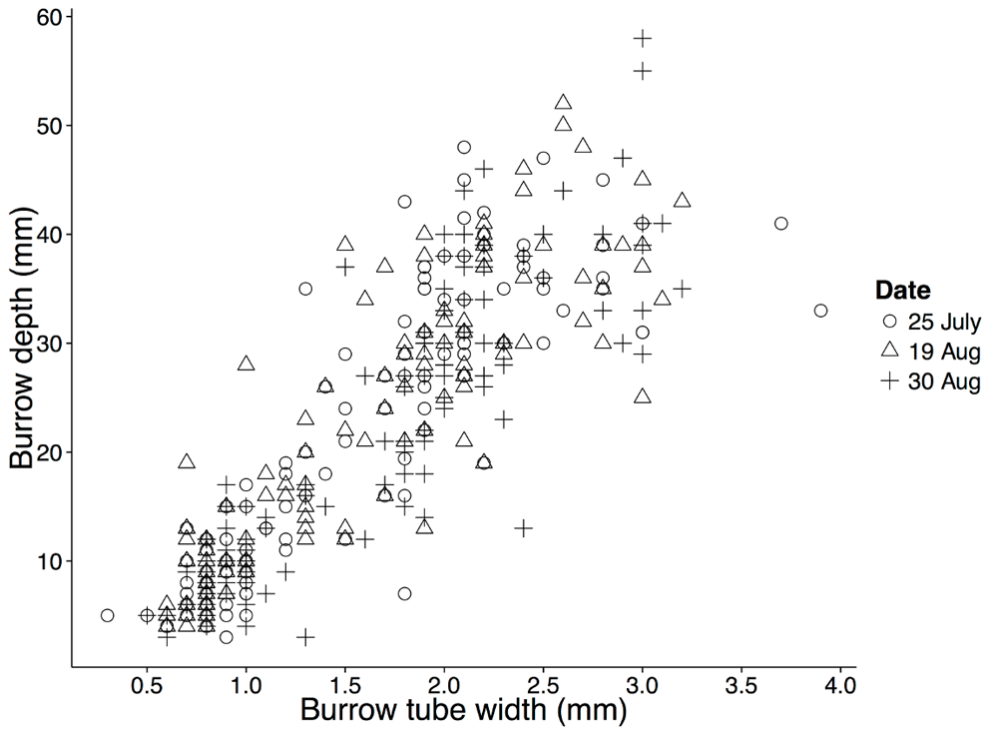
Depth (mm) versus tube width (mm) of *Corophium volutator* burrows in Pecks Cove in summer 2011. Burrow measurements were taken from polymer resin casts of naturally occurring burrows on three sampling dates, with n = 3, 5 and 6 casts, respectively. These dates were selected to roughly coincide with before arrival, peak abundance and departure of shorebirds. The linear regression coefficients for depth (mm) vs. width (mm) of burrows are 15.4±0.5 (SE) for the slope and −2.7±0.9 for the intercept.

Depth of *C. volutator* burrows increased with tube width (linear regression, adjusted R^2^ = 0.75, df = 340, p<0.0001, Figure 5). However, once variation in width was accounted for, burrow depth did not vary with date (ANOVA, F_2,6_ = 0.26, p = 0.78). The maximum burrow depth measured was 58 mm. The *C. volutator* population structure at Pecks Cove was generally similar among sampling dates (Figure S4).

### Predator simulation experiment

**Figure 6:**
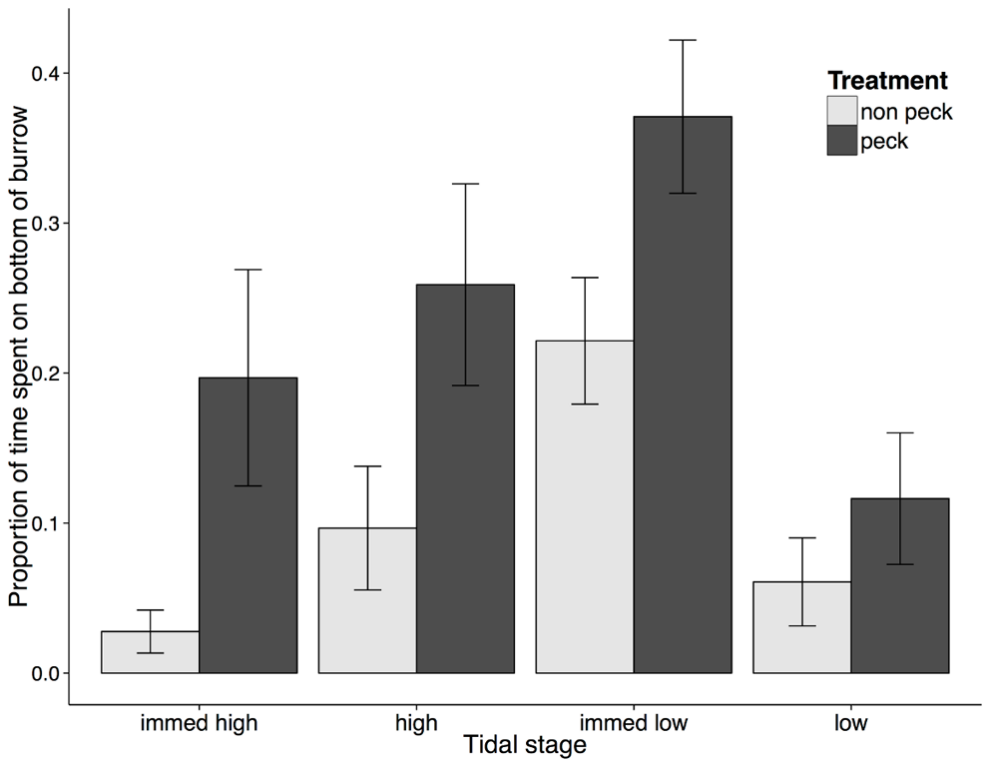
Proportion of time spent by focal *Corophium volutator* females on the bottom of their burrows in the presence and absence of simulated shorebird predation in laboratory. Clustered bars represent paired treatment levels (pecking and non-pecking) for each of four simulated tidal stages (immediately high, high, immediately low, and low). Proportion of time was calculated from 80 min of observation time per experimental unit and tidal stage. Experimental units in the pecking treatment level experienced 5 min of simulated pecking (1 peck per s) at the start of immediately low tide. Values are mean ±1 SE (n = 6 experimental units).

*C. volutator* females spent significantly more time on the bottom of their burrows when exposed to simulated shorebird pecking compared to controls without pecking (F_1,10_ = 7.62, p = 0.02, Figure 6). This effect did not vary with tidal stage (treatment x tide interaction, F_3,30_ = 0.87, p = 0.47). Even though amphipods were exposed to the predator simulation for only the first 5 minutes of low tide, the effect persisted throughout low and high tide. However, time spent on the bottom of a burrow did vary with tide (F_3,30_ = 11.6, p<0.0001); *C. volutator* females spent the greatest amount of time at the bottom of their burrows during immediately low tide. Mean (±SD) burrow depth was 18.4±6.7 mm and 16.4±7.5 mm for the non-pecking and pecking treatment levels, respectively.

## Discussion

Predator avoidance strategies can affect the outcome of predator-prey interactions [19], [74], [75], [76]. For example, if prey relocate to an area with reduced risk of detection and capture [74], the result is a reversible decline in prey availability. Predator avoidance in shorebird-*Corophium volutator* interactions has been primarily identified through cessation of crawling where shorebirds are present and behavioral responses of the predator [54], [57], [77], [78], [79]. By quantifying vertical position in the sediment in the presence of predators (or simulated predators), our study provides direct evidence that *C. volutator* in the Bay of Fundy employ a predator avoidance mechanism beyond simple cessation of crawling.

### Seasonal trends versus predator effects

In our predator exclusion experiment, we observed a distinct, temporal vertical redistribution of adult *C. volutator*. Although amphipods moved deeper into the sediment later in the summer, which happens to correspond with arrival of shorebirds, this redistribution was detectably stronger in areas where birds were able to forage compared to areas where birds were excluded. We also observed a concurrent cessation of crawling activity by amphipods, consistent with previous findings in the Bay of Fundy [46]. The observed vertical shift in the sediment was not simply due to selective predation of amphipods on or near the sediment surface, because absolute adult abundances in deeper layers increased after birds arrived and in areas where birds were able to forage, while the overall number of adults remained relatively consistent throughout the experiment (Figure 3). Sediment temperature was also not associated with the observed redistribution. High sediment temperatures can cause *C. volutator* to retreat deeper into burrows [80]; however, the highest temperature we recorded was on 29 July, after birds arrived but before they began foraging in flocks and prior to the most obvious redistribution by amphipods. Moreover, sediment temperatures in our study were lower than those tested by Gilroy [80].

In the field it is difficult to separate seasonal and bird effects to establish a causal relationship on vertical redistribution of infaunal prey. However, using appropriate controls in the field we did detect a significant difference in vertical distribution between areas with and without shorebird foraging activity on both August sampling dates for small *C. volutator* adults and on 8 August for large *C. volutator* adults. Further, the results of our predator simulation experiment corroborate our field observations; in the presence of perceived predator activity, *C. volutator* individuals spent more time deeper in their burrows. Minderman et al. [55] found that amphipod prey retreated into burrows in response to increased predation risk. Similarly, various species of bivalves have been shown to increase survival by burrowing deeper into the sediment in response to predators [19], [76]. Our data suggest that *C. volutator* adults respond to the risk of predation, both by ceasing crawling and burrowing into the sediment beyond the reach of probing sandpipers.

### Predator avoidance mechanisms

To avoid predation, *C. volutator* individuals could occupy lower portions of the sediment either by moving lower into existing burrows or by constructing deeper burrows. We found that C. *volutator* burrow depth did not increase later in the season when shorebirds were present, nor was it deeper for *C. volutator* exposed to simulated predator activity than those in the non-pecking treatment of the lab experiment. Rather, amphipods increased time spent at the bottom of their burrows in the presence of simulated predation risk. However, individuals would not necessarily have to go all the way to the bottom of their burrow to evade a predator. We observed that adult *C. volutator* burrows extend to ∼6 cm in the sediment at Pecks Cove, while depths of up to 10 cm have been reported in the literature [30]. Sandpipers foraging in the Bay of Fundy have bills ∼20 mm in length [50]; thus, it would be difficult for sandpipers to obtain prey burrowed deeper than 2 cm. Based on this, time at the bottom of a burrow for amphipods occupying deep burrows is a conservative metric for quantifying predator avoidance. An amphipod would not necessarily need to go to the bottom of its burrow to avoid a predator; even slight adjustments in its depth could reduce the risk of being eaten [81].

### Trade-off between foraging and predation risk

Although we observed a vertical redistribution of prey, we still found amphipods in the top sediment layer in our predator exclusion experiment. This incomplete predator avoidance response is probably related to the fact that prey must deal with the competing pressures of avoiding predation and foraging for growth [82], [83], [84]. To manage this growth/predation risk trade-off, prey must make decisions regarding allocation of time to foraging and antipredator behavior [82], [85], [86]. Sandpipers forage with consistent success rates throughout low tide [87], [88] and *C. volutator* individuals would presumably not be able to avoid foraging for that entire period. For *C. volutator*, food access is better near the sediment surface [29], [31], [89], which may explain why, in spite of the increased predation risk, we observed individuals near the top of their burrows.

### Spatial scale and magnitude of the response by *C. volutator*

*C. volutator* may be able to broadly detect and respond to predators. This is suggested by our observation that, in addition to the response in areas exposed to predation, *C. volutator* individuals in bird exclosures also reduced crawling and appeared deeper in the sediment when shorebirds were present compared to before their arrival. However, based on our predator exclusion experiment, *C. volutator*'s response is stronger when directly exposed to foraging shorebirds. Stillman et al. [79], in modeling predator avoidance in a *C. volutator-*Redshank system based on observations by Yates et al. [54], estimated that *C. volutator* individuals respond to shorebirds within a 0.6-m radius (range 0.1 to 1 m). Thus, shorebirds foraging within our study area could potentially be detected by *C. volutator* in an exclosure, and so it is not surprising that we also observed a response in our exclosures. In addition, *C. volutator* exhibit considerable swimming movement during high tide [31], [90], [91], and could conceivably redistribute among bird exclosures and control plots. We observed that the effect of a perceived predator persisted throughout the tidal cycle in our lab experiment. Thus, amphipods collected from bird exclosures could have been responding to previously experienced predation risk. Given all of these factors, which could minimize differences between exclosures and control plots, our field experiment was a conservative test of predator avoidance. Indeed, we suggest that observing treatment effects on vertical distribution at the scale we tested is indicative of a strong response. Additional experiments with larger exclosures and/or enclosures to restrict emigration of swimming *C. volutator* are required to identify the scale of the response and to isolate the cues that trigger vertical redistribution and cessation of crawling.

### Implications and future work

Prey availability for Semipalmated Sandpipers is typically assessed based on absolute density of *C. volutator* [46], [92], [93], [94]. However, recent work on foraging behavior [95] and stable isotopes [42] indicates that sandpipers staging in the Bay of Fundy actually have a much broader diet. Our study suggests that traditional methods of sampling prey may misrepresent actual availability, which could contribute to the recent observation that *C. volutator* comprises a smaller portion of the sandpiper's diet than previously assumed [42]. As Bay of Fundy mudflats represent critical stopover habitat for migrating shorebirds [40], [96], an accurate estimate of prey availability is needed to understand habitat use. Future studies in this system and others should consider vertical distribution of prey.

The behavioral response to predation we have observed in *C. volutator* could have community-wide implications. Predator avoidance limits prey availability [13], [52], [53] and, among shorebird-prey interactions, has been linked to changes in feeding [57] and activity rates [55], as well as interference competition among foraging conspecifics [54], [55], [79]. By burrowing out of reach of shorebird predators, *C. volutator* could displace predation pressure onto other, more accessible, prey items as has been observed in burrowing bivalves [19]. It has also been suggested that when foraging shorebirds increase activity rates in response to behavioral changes in their prey, they themselves experience a heightened risk of predation [55]. The non-consumptive effects of predation on community dynamics are considerable [7]. Studies of predator avoidance strategies are a key element to understanding the cascading impacts of predator-prey interactions.

## Supporting Information

Figure S1 **Experimental setup of the laboratory experiment examining effects of simulated shorebird predation on** ***Corophium volutator*** **behavior.** Pictures showing the A) laboratory set up, B) thin glass cages with cedar frame used to observed behaviors of *Corophium volutator* adults in their burrow and C) burrows with a *C. volutator* individual inside, in one of the glass cages.
(DOCX)

Figure S2 **S2.1: Proportion of time** ***Corophium volutator*** **individuals spent in each of seven behaviors while in their burrow in the presence and absence of simulated shorebird pecking in a laboratory experiment.** We monitored behavior over the course of a tidal cycle (20-min observation periods in each tidal stage per day conducted over 4 days; tidal stages: immediately high, high, immediately low, and low). To mimic natural conditions of shorebird predation, pecking occurred for the first 5 min of immediately low tide at a rate of one peck per s. Each section of a stack represents the mean proportion of time spent engaged in the corresponding behavior. Stacks do not sum to 1, because individuals were not always visible for behavior to be recorded. Error bars represent ±1 SE, n = 6 experimental units. **S2.2: Counts per 20 min of observation that** ***Corophium volutator*** **individuals spent in each of five types of movement in the presence and absence of simulated shorebird pecking in a laboratory experiment.** We monitored behavior over the course of a tidal cycle (20-min observation periods in each tidal stage per day conducted over 4 days; tidal stages: immediately high, high, immediately low, and low). To mimic natural conditions of shorebird predation, pecking occurred for the first 5 min of immediately low tide at a rate of one peck per s. Bars represent the mean number of times *C. volutator* individuals were observed engaging in a particular movement; error bars represent ±1 SE, n = 6 experimental units.
(DOCX)

Figure S3 **Proportional vertical distribution of** ***Corophium volutator*** **adults sampled from the predator exclusion experiment at Pecks Cove in 2007.** Proportion of *C. volutator* sized A) 4–6 mm and B) >6 mm in bird exclosures (−B) and control plots (+B) found in each vertical layer of sediment. Layers 1–4 are 0–5, 0.5–1.5, 1.5–3.0, and 3.0–5.0 cm from the sediment surface, respectively.
(DOCX)

Figure S4 **Population structure of** ***Corophium volutator*** **at Pecks Cove in summer 2011.** Sample sizes represent the number of individuals measured (pooling over cores) to generate the size frequency distribution in each sampling round.
(DOCX)

Data S1 **Raw data from three studies examining predator avoidance behavior of adult** ***Corophium volutator*** **in the presence of shorebirds.**
(XLSX)

## References

[1] Paine RT (1966) Food web complexity and species diversity. Am Nat 100: 65–75.

[2] Menge BA (1976) Organization of the New England rocky intertidal community: role of predation, competition and environmental heterogeneity. Ecol Monogr 46: 355–393.

[3] Menge BA, Sutherland JP (1976) Species diversity gradients: synthesis of the roles of predation, competition, and temporal heterogeneity. Am Nat 110: 351–369.

[4] Huntly N (1991) Herbivores and the dynamics of communities and ecosystems. Ann Rev Ecol Syst 22: 477–503.

[5] Vieira EA, Duarte LFL, Dias GM (2012) How the timing of predation affects composition and diversity of species in a marine sessile community? J Exp Mar Biol Ecol 412: 126–133.

[6] Werner EE, Peacor SD (2003) A review of trait-mediated indirect interactions in ecological communities. Ecology 84: 1083–1100.

[7] Preisser EL, Bolnick DI, Benard MF (2005) Scared to death? The effects of intimidation and consumption in predator-prey interactions. Ecology 86: 501–509.

[8] Paterson RA, Pritchard DW, Dick JTA, Alexander ME, Hatcher MJ, et al (2013) Predator cue studies reveal strong trait-mediated effects in communities despite variation in experimental designs. Anim Behav 86: 1301–1313.

[9] Lindquist N, Hay ME (1996) Palatability and chemical defense of marine invertebrate larvae. Ecol Monogr 66: 431–450.

[10] Honma A, Oku S, Nishida T (2006) Adaptive significance of death feigning posture as a specialized inducible defence against gape-limited predators. Proc Biol Sci 273: 1631–1636. 16769634 10.1098/rspb.2006.3501 PMC1634928

[11] Domenici P, Turesson H, Brodersen J, Brönmark C (2008) Predator-induced morphology enhances escape locomotion in crucian carp. Proc Biol Sci 275: 195–201. 17971327 10.1098/rspb.2007.1088 PMC2596180

[12] Manzur T, Vidal F, Pantoja JF, Fernandez M, Navarrete SA (2014) Behavioural and physiological responses of limpet prey to a seastar predator and their transmission to basal trophic levels. J Anim Ecol doi: 10.1111/1365–2656.12199. 10.1111/1365-2656.12199 24428576

[13] Backwell PRY, O'Hara PD, Christy JH (1998) Prey availability and selective foraging in shorebirds. Anim Behav 55: 1659–1667. 9642009 10.1006/anbe.1997.0713

[14] Menge BA, Lubchenco J (1981) Community organization in temperature and tropical rocky intertidal habitats: prey refuges in relation to consumer pressure gradients. Ecol Monogr 51: 429–450.

[15] Meerhoff M, Iglesias C, De Mello FT, Clemente JM, Jenson E, et al (2007) Effects of habitat complexity on community structure and predator avoidance behavior of littoral zooplankton in temperate versus subtropical shallow lakes. Freshw Biol 52: 1009–1021.

[16] Wilson WH (1990) Competition and predation in marine soft-sediment communities. Ann Rev Ecol Syst 21: 221–241.

[17] Holohan BA, Klos EG, Oviatt CA (1998) Population density, prey selection, and predator avoidance of the burrowing anemone (*Ceriantheopsis americanus*) in Narragansett Bay, Rhode Island. Estuaries 21: 466–469.

[18] Tallqvist M (2001) Burrowing behaviour of the Baltic clam *Macoma balthica*: effects of sediment type, hypoxia and predator presence. Mar Ecol Prog Ser 212: 183–191.

[19] Griffiths CL, Richardson CA (2006) Chemically induced predator avoidance behaviour in the burrowing bivalve *Macoma balthica* . J Exp Mar Biol Ecol 331: 91–98.

[20] Dill LM, Fraser AHG (1997) The worm re-turns: hiding behaviour of a tube-dwelling marine polychaete, *Serpula vermicularis* . Behav Ecol 8: 186–193.

[21] Kicklighter CE, Hay ME (2007) To avoid or deter: interactions among defensive and escape strategies in sabellid worms. Oecologia 151: 161–173. 17136395 10.1007/s00442-006-0567-0

[22] Peer DL, Linkletter LE, Hicklin PW (1986) Life history and reproductive biology of *Corophium volutator* (Crustacea: Amphipoda) and the influence of shorebird predation on population structure in Chignecto Bay, Bay of Fundy, Canada. Netherlands J Sea Res 20: 359–373.

[23] Barbeau MA, Grecian LA, Arnold EE, Sheahan DC, Hamilton DJ (2009) Spatial and temporal variation in the population dynamics of the intertidal amphipod *Corophium volutator* in the upper Bay of Fundy, Canada. J Crustacean Biol 29: 491–506.

[24] Boates JS, Smith PC (1989) Crawling behaviour of the amphipod *Corophium volutator* and foraging by Semipalmated Sandpipers, *Calidris pusilla* . Can JZool 67: 457–462.

[25] Vader W (1964) A preliminary investigation into the reactions of the infauna of the tidal flats to tidal fluctuations in water level. Netherlands J Sea Res 2: 189–222.

[26] Mills A, Fish JD (1980) Effects of salinity and temperature on *Corophium volutator* and *C. arenarium* (Crustacea: Amphipoda), with particular reference to distribution. Mar Biol 58: 153–161.

[27] Mathews SL, Boates JS, Walde SJ (1992) Shorebird predation may cause discrete generations in an amphipod prey. Ecography 15: 393–400.

[28] Limia J, Raffaelli D (1997) The effects of burrowing by the amphipod *Corophium volutator* on the ecology of intertidal sediments. J Mar Biol Assoc UK 77: 409–423.

[29] De Backer A, Van Ael E, Vincx M, Degraer S (2010) Behaviour and time allocation of the mud shrimp, *Corophium volutator*, during the tidal cycle: a laboratory study. Helgol Mar Res 64: 63–67.

[30] McCurdy DG, Boates JS, Forbes MR (2000) Reproductive synchrony in the intertidal amphipod *Corophium volutator* . Oikos 88: 301–308.

[31] Meadows PS, Reid A (1966) The behaviour of *Corophium volutator* (Crustacea: Amphipoda). J Zool Lond 150: 387–399.

[32] Fish JD, Mills A (1979) The reproductive biology of *Corophium volutator* and *C. arenarium* (Crustacea: Amphipoda). J Mar Biol Assoc UK 59: 355–368.

[33] Forbes MR, Boates SJ, McNeil NL, Brison AE (1996) Mate searching by males of the intertidal amphipod *Corophium volutator* (Pallas). Can J Zool 74: 1479–1484.

[34] Lawrie SM, Raffaelli DG (1998) In situ swimming behaviour of the amphipod *Corophium volutator* (Pallas). J Exp Mar Biol Ecol 224: 237–251.

[35] Raffaelli D, Milne H (1987) An experimental investigation of the effects of shorebird and flatfish predation on estuarine invertebrates. Estuar Coast Shelf Sci 24: 1–13.

[36] McCurdy DG, Forbes MR, Logan SP, Lancaster D, Mautner SI (2005) Foraging and impacts by benthic fish on intertidal amphipod *Corophium volutator* . J Crustacean Biol 25: 558–564.

[37] Cheverie AV, Hamilton DJ, Coffin MRS, Barbeau MA (2014) Effects of shorebird predation and snail abundance on an intertidal mudflat community. J Sea Res, in press.

[38] Coulthard ME, Hamilton DJ (2011) Effects of *Ilyanassa obsoleta* (Say) on the abundance and vertical distribution of *Corophium volutator* (Pallas) on mudflats of the upper Bay of Fundy. J Exp Mar Biol 397: 161–172.

[39] Coffin MRS, Barbeau MA, Hamilton DJ, Drolet D (2012) Effect of the mud snail *Ilyanassa obsoleta* on vital rates of the intertidal amphipod *Corophium volutator* . J Exp Mar Biol Ecol 418: 12–23.

[40] Hicklin PW (1987) The migration of shorebirds in the Bay of Fundy. Wilson Bull 99: 540–570.

[41] Neima SG (2014) Movement patterns, duration of stay, and diet of Semipalmated Sandpipers (*Calidris pusilla*) during migratory stopover in the upper Bay of Fundy. BSc Honours Thesis. Mount Allison University, Sackville, New Brunswick, Canada.

[42] Quinn JT, Hamilton DJ (2012) Variation in diet of Semipalmated Sandpipers (*Calidris pusilla*) during stopover in the upper Bay of Fundy, Canada. Can J Zool 90: 1181–1190.

[43] Boates JS (1980) Foraging Semipalmated Sandpipers *Calidris pusilla* L., and their major prey, *Corophium volutator* (Pallas) on the Starrs Point mudflat, Minas Basin. MSc Thesis, Acadia University, Wolfville, Nova Scotia, Canada.

[44] Stoddard PK, Marsden JE, Williams TC (1983) Computer simulation of autumnal bird migration over the western North Atlantic. Anim Behav 31: 173–180.

[45] Hicklin PW, Smith PC (1984) Selection of foraging sites and invertebrate prey by migrant Semipalmated Sandpipers, *Calidris pusilla* (Pallas), in Minas Basin, Bay of Fundy. Can J Zool 62: 2201–2210.

[46] Boates JS, Forbes M, Zinck M, McNeil N (1995) Male amphipods (*Corophium volutator* [Pallas]) show flexible behaviour in relation to risk of predation by sandpipers. Ecoscience 2: 123–128.

[47] Goss-Custard JD (1970) The responses of Redshank (*Tringa totanus* (L.)) to spatial variations in the density of their prey. J Anim Ecol 39: 91–113.

[48] Gratto GW, Thomas MLH, Gratto CL (1984) Some aspects of the foraging ecology of migrant juvenile sandpipers in the outer Bay of Fundy. Can J Zool 62: 1889–1892.

[49] Durell SEA (2000) Individual feeding specialisation in shorebirds: population consequences and conservation implications. Biol Rev 75: 503–518. 11117199 10.1111/j.1469-185x.2000.tb00053.x

[50] Gratto-Trevor CL, Morrison RIG, Mizrahi D, Lank DB, Hicklin P, et al (2012) Migratory connectivity of Semipalmated Sandpipers: winter distribution and migration routes of breeding populations. Waterbirds 35: 83–95.

[51] Hill C, Elmgren R (1987) Vertical distribution in the sediment in the co-occurring benthic amphipods *Pontoporeia affinis* and *P. femorata* . Oikos 49: 221–229.

[52] Charnov EL, Orians GH, Hyatt K (1976) Ecological implications of resource depression. Am Nat 110: 247–259.

[53] Goss-Custard JD (1980) Competition for food and interference among waders. Ardea 68: 31–52.

[54] Yates MG, Stillman RA, Goss-Custard JD (2000) Contrasting interference functions and foraging dispersion in two species of shorebird (Charadrii). J Anim Ecol 69: 314–322.

[55] Minderman J, Lind J, Cresswell W (2006) Behaviourally mediated indirect effects: interference competition increases predation mortality in foraging redshanks. J Anim Ecol 75: 713–723. 16689954 10.1111/j.1365-2656.2006.01092.x

[56] Robar NDP (2006) Fine-scale effects of prey density and interference on Semipalmated Sandpipers (*Calidris pusilla*) in the upper Bay of Fundy. BSc Honours Thesis, Mount Allison University, Sackville, New Brunswick, Canada.

[57] Beauchamp G (2007) Competition in foraging flocks of migrating semipalmated sandpipers. Oecologia 154: 403–409. 17676344 10.1007/s00442-007-0818-8

[58] Hamilton DJ, Diamond AW, Wells PG (2006) Shorebirds, snails, and the amphipod (*Corophium volutator*) in the upper Bay of Fundy: top-down vs. bottom-up factors, and the influence of compensatory interactions on mudflat ecology. Hydrobiologia 567: 285–306.

[59] Boudreau MR, Hamilton DJ (2012) Seasonal variation in effects of multiple predators on an intertidal mussel bed: implications for interpretation of manipulative experiments. Mar Ecol Prog Ser 465: 137–153.

[60] Robar NDP, Hamilton DJ (2007) A method for estimating habitat use by shorebirds using footprints. Waterbirds 30: 116–120.

[61] Crewe TL, Hamilton DJ, Diamond AW (2001) Effects of mesh size on sieved samples of *Corophium volutator* . Estuar Coast Shelf Sci 53: 151–154.

[62] Wallace EH (2008) Effects of predation by Semipalmated Sandpipers (*Calidris pusilla*) on vertical distribution of the amphipod *Corophium volutator.* BSc Honours Thesis. Mount Allison University, Sackville, New Brunswick, Canada.

[63] Gingras MK, Pickerill R, Pemberton SG (2002) Resin cast of modern burrows provides analogs for composite trace fossils. Palaios 17: 206–211.

[64] Drolet D, Barbeau MA (2012) Population structure of resident, immigrant, and swimming *Corophium volutator* (Amphipoda) on an intertidal mudflat in the Bay of Fundy, Canada. J Sea Res 70: 1–13.

[65] Campbell JI, Meadows PS (1974) Gregarious behavior in a benthic marine amphipod (*Corophium volutator*). Experientia 30: 1396–1397.

[66] SPSS Inc. (2006) SPSS version 16.0. SPSS, Chicago, Illinois.

[67] R Development Core Team (2009) R: A language and environment for statistical computing. R Foundation for Statistical Computing, Vienna, Austria. Available: https://www.R-project.org. Accessed 8 October 2014.

[68] Leech NL, Barrett KC, Morgan GA (2008) SPSS for Intermediate Statistics: use and interpretation, 3rd ed. New York: Taylor and Francis Group. 270 p.

[69] Norušis MJ (2008) SPSS 16.0 advanced statistical procedures companion. New Jersey: Prentice Hall. 418 p.

[70] Keppel G (1991) Design and analysis: a researcher's handbook, 3rd ed. New Jersey: Prentice-Hall. 594 p.

[71] Zar JH (1999) Biostatistical analysis, 4th ed. New Jersey: Prentice Hall. 663 p.

[72] Burnham JS, Anderson DR (2002) Model selection and multi-model inference: a practical information-theoretic approach, 2^nd^ ed. New York: Springer. 488 p.

[73] Winer BJ, Brown DR, Michels KM (1991) Statistical Principles in Experimental Design, 3rd ed. Boston: McGraw Hill. 1057 p.

[74] Zwarts L, Esselink P (1989) Versatility of male curlews *Numenius arquata* preying upon *Nereis diversicolor*: deploying contrasting capture modes dependent on prey availability. Mar Ecol Prog Ser 56: 255–269.

[75] Kruse I, Buhs F (2000) Preying at the edge of the sea: the nemertine *Tetrastemma melanocephalum* and its amphipod prey on high intertidal sandflats. Hydrobiologia 426: 43–55.

[76] Flynn AM, Smee DL (2010) Behavioral plasticity of the soft-shell clam, *Mya arenaria* (L.), in the presence of predators increases survival in the field. J Exp Mar Biol Ecol 383: 32–38.

[77] Goss-Custard JD (1977) Predator responses and prey mortality in Redshank, *Tringa totanus* (L.), and a preferred prey, *Corophium volutator* (Pallas). J Anim Ecol 46: 21–35.

[78] Selman J, Goss-Custard JD (1988) Interference between foraging redshanks *Tringa totanus* . Anim Behav 36: 1542–1544.

[79] Stillman RA, Goss-Custard JD, Alexander MJ (2000) Predator search pattern and the strength of interference through prey depression. Behav Ecol 11: 597–605.

[80] Gilroy CE (2012) The effect of temperature and tidal exposure on behaviour and survival of *Corophium volutator*. BSc Honours Thesis, Mount Allison University, Sackville, New Brunswick, Canada.

[81] Myers JP, Williams SL, Pitelka FA (1980) An experimental analysis of prey availability for sanderlings (Aves: Scolopacidae) feeding on sandy beach crustaceans. Can J Zool 58: 1564–1574.

[82] Lima SL, Dill LM (1990) Behavioral decisions made under the risk of predation: a review and prospectus. Can J Zool 68: 619–640.

[83] Werner EE, Anholt BR (1993) Ecological consequences of the trade-off between growth and mortality rates mediated by foraging activity. Am Nat 142: 242–272. 19425978 10.1086/285537

[84] McPeek MA (2004) The growth/predation risk trade-off: so what is the mechanism? Am Nat 163: E88–E111. 15122497 10.1086/382755

[85] Brodin T, Johansson F (2004) Conflicting selection pressures on the growth/predation-risk trade-off in a damselfly. Ecology 85: 2927–2932.

[86] Strobbe F, McPeek MA, De Block M, Stoks R (2011) Fish predation selects for reduced foraging activity. Behav Ecol Sociobiol 65: 241–247.

[87] Beauchamp G (2005) Low foraging success of Semipalmated Sandpipers at the edges of groups. Ethology 111: 785–798.

[88] Beauchamp G (2006) Spatial, temporal and weather factors influencing the foraging behavior of migrating Semipalmated Sandpipers. Waterbirds 29: 221–225.

[89] Riisgård HU, Schotge P (2007) Surface deposit-feeding versus filter-feeding in the amphipod *Corophium volutator* . Mar Biol Res 3: 421–427.

[90] Drolet D, Barbeau MA (2009) Diel and semi-lunar cycles in the swimming activity of the intertidal, benthic amphipod *Corophium volutator* in the upper Bay of Fundy, Canada. J Crustacean Biol 29: 51–56.

[91] Bringloe TT, Drolet D, Barbeau MA, Forbes MR, Gerwing TG (2013) Spatial variation in population structure and its relation to movement and the potential for dispersal in a model intertidal invertebrate. PLoS ONE 8(7): e69091 doi:10.1371/journal.pone.0069091 23874877 10.1371/journal.pone.0069091 PMC3709997

[92] Wilson WH (1989) Predation and the mediation of intraspecific competition in an infaunal community in the Bay of Fundy. J Exp Mar Biol Ecol 132: 221–245.

[93] Hamilton DJ, Barbeau MA, Diamond AW (2003) Shorebirds, mud snails, and *Corophium volutator* in the upper Bay of Fundy, Canada: predicting bird activity on intertidal mudflats. Can J Zool 81: 1358–1366.

[94] Beauchamp G (2009) Functional response of staging semipalmated sandpipers feeding on burrowing amphipods. Oecologia 161: 651–655. 19543917 10.1007/s00442-009-1398-6

[95] MacDonald EC, Ginn MG, Hamilton DJ (2012) Variability in foraging behavior and implications for diet breadth among Semipalmated Sandpipers staging in the upper Bay of Fundy. Condor 114: 135–144.

[96] Hicklin PW, Chardine JW (2012) The morphometrics of migrant Semipalmated Sandpipers in the Bay of Fundy: evidence for declines in the eastern breeding population. Waterbirds 35: 74–82.

