# Supplementary material for: Behavioral Response of Corophium volutator to Shorebird Predation in the Upper Bay of Fundy, Canada
Source: PLoS One. 2014 Oct 29;9(10):e110633. doi: 10.1371/journal.pone.0110633 (PMC4212999; doi:10.1371/journal.pone.0110633)
Supplement: Figure S1 — Experimental setup of the laboratory experiment examining effects of simulated shorebird predation on Corophium volutator behavior. Pictures showing the A) laboratory set up, B) thin glass cages with cedar frame used to observed behaviors of Corophium volutator adults in their burrow and C) burrows with a C. volutator individual inside, in one of the glass cages. (DOCX) [file pone.0110633.s001.docx]

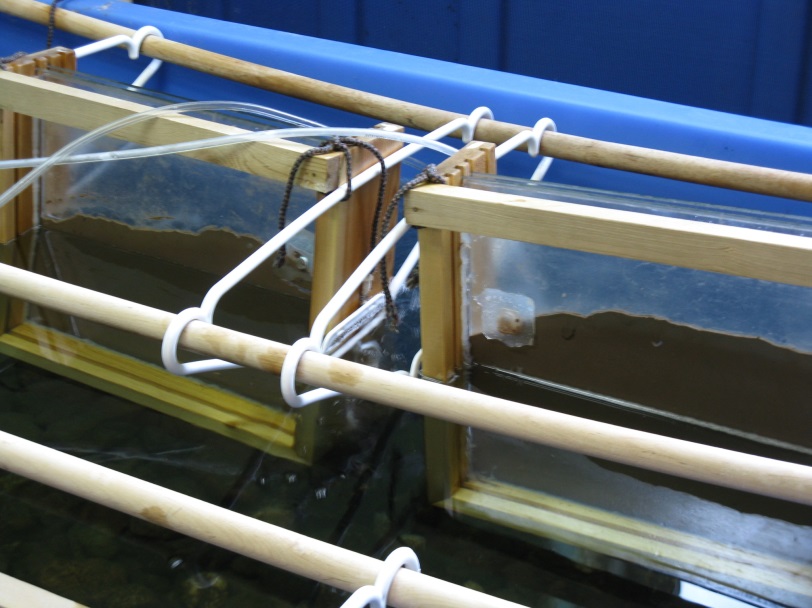

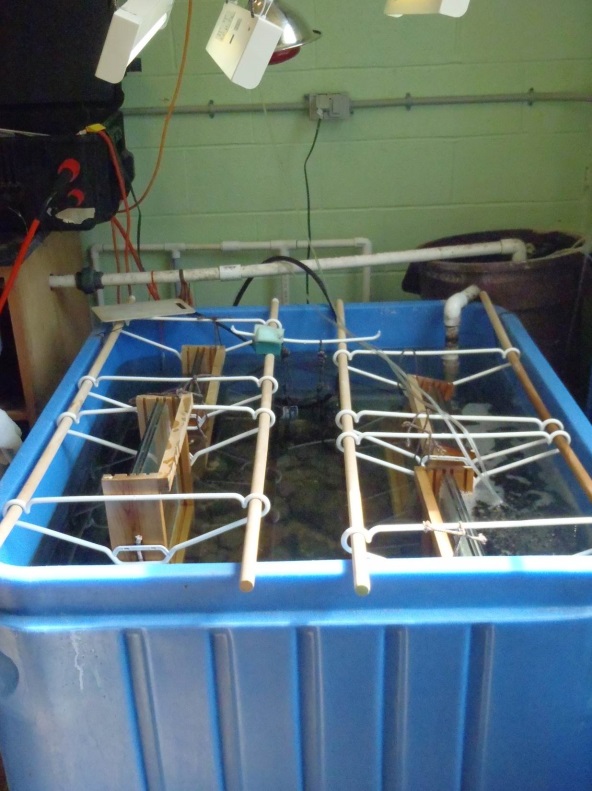

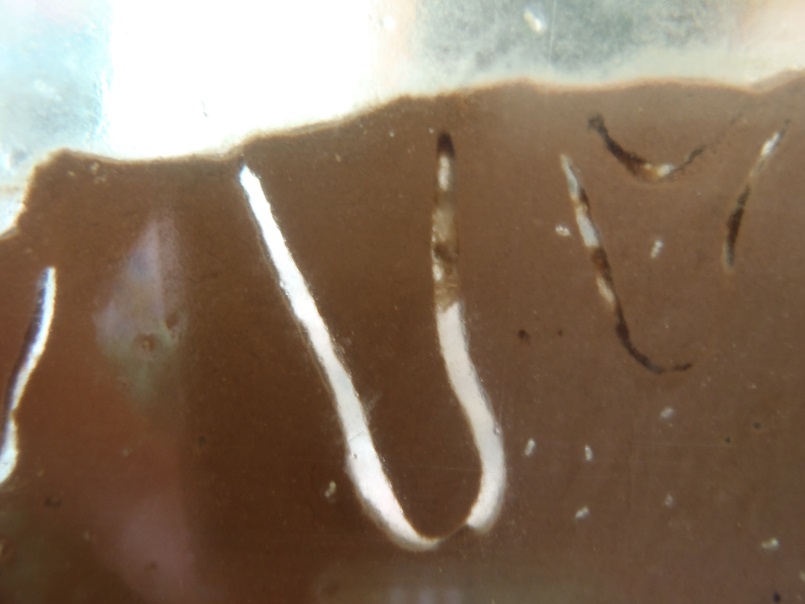


10 mm

A)

B)

C)

**Figure S1. Experimental setup of the laboratory experiment examining effects of simulated shorebird predation on *Corophium volutator* behavior.**

Pictures showing the A) laboratory set up, B) thin glass cages with cedar frame used to observed behaviors of *Corophium volutator* adults in their burrow and C) burrows with a *C. volutator* individual inside in one of the glass cages.
