# Supplementary material for: Behavioral Response of Corophium volutator to Shorebird Predation in the Upper Bay of Fundy, Canada
Source: PLoS One. 2014 Oct 29;9(10):e110633. doi: 10.1371/journal.pone.0110633 (PMC4212999; doi:10.1371/journal.pone.0110633)
Supplement: Figure S2 — S2.1: Proportion of time Corophium volutator individuals spent in each of seven behaviors while in their burrow in the presence and absence of simulated shorebird pecking in a laboratory experiment. We monitored behavior over the course of a tidal cycle (20-min observation periods in each tidal stage per day conducted over 4 days; tidal stages: immediately high, high, immediately low, and low). To mimic natural conditions of shorebird predation, pecking occurred for the first 5 min of immediately low tide at a rate of one peck per s. Each section of a stack represents the mean proportion of time spent engaged in the corresponding behavior. Stacks do not sum to 1, because individuals were not always visible for behavior to be recorded. Error bars represent ±1 SE, n = 6 experimental units. S2.2: Counts per 20 min of observation that Corophium volutator individuals spent in each of five types of movement in the presence and absence of simulated shorebird pecking in a laboratory experiment. We monitored behavior over the course of a tidal cycle (20-min observation periods in each tidal stage per day conducted over 4 days; tidal stages: immediately high, high, immediately low, and low). To mimic natural conditions of shorebird predation, pecking occurred for the first 5 min of immediately low tide at a rate of one peck per s. Bars represent the mean number of times C. volutator individuals were observed engaging in a particular movement; error bars represent ±1 SE, n = 6 experimental units. (DOCX) [file pone.0110633.s002.docx]

**Supporting Information 2.** **Effect of simulated pecking on behavior and movement of *Corophium volutator***

In our predator simulation experiment in the laboratory, we observed behavior and movement of *Corophium volutator* females in the focal burrows to determine if the simulated pecking treatment level had confounding behavioral effects. See methods in the paper for details on experimental design.

Burrow behavior: *C. volutator* individuals spent 85.5 ± 10.7% and 93.2 ± 5.3% of their time in their burrow in the non-pecking and pecking treatment levels, respectively (out of 80 min of observation time per experimental unit-tidal stage combination). While in the burrow, we observed amphipods engaging in seven behaviors: still, fluttering pleopods, curled body, antennae in water, antennae scraping the surface of the mud, interacting with other individuals, and burrow maintenance. An individual was considered still when it remained motionless in the burrow. Behavior with fluttering pleopods was characterized by rapid movements of abdominal appendages. An individual was considered curled when the end of the telson was brought up to the rostrum to form a tight ball. Antennae in water occurred when an individual was at the top of its burrow with its antennae erect in the water column. Antennae scraping occurred when an individual was at the opening of its burrow, using antennae to scrape the surface of the mud, bring material into its burrow. An interacting individual was recorded during any sort of touching between it and another one or more individuals. Finally, burrow maintenance was characterized by an individual pushing mud out of its burrow or packing mud against the walls of its burrow. For each experimental unit-tidal stage combination, we calculated the proportion of time an individual engaged in each of these behaviors out of the time in the burrow.

Individuals were generally still or fluttering their pleopods, a trend largely consistent among treatment levels and tidal stages (Figure S1.2). Fluttering pleopods has been associated with filter feeding and aerating the burrow by creating currents through the U-shaped tube [1]. Burrow maintenance occurred more often in experimental units with pecking compared to those without pecking. The top of burrows in the experimental units being pecked had a tendancy to collapse; therefore, it is most likely that maintenance is done to strengthen the burrow, and occurs only when needed. Still, burrow maintenance accounted for a minimal portion of the time budget.

Movement: *C. volutator* individuals engaged in five types of movements: entering a burrow, exiting a burrow, moving up and down within a burrow, somersaulting (changing direction) and zipping across the bottom of the burrow (from one burrow opening to the other opening). Individuals moved up and down in the burrow most frequently, and movement patterns did not differ among treatment levels (Figure S1.3).

Conclusion: Overall, we did not observe any large differences in burrow behavior or movement among treatment levels, and conclude that our simulated pecking did not cause abnormal stress.


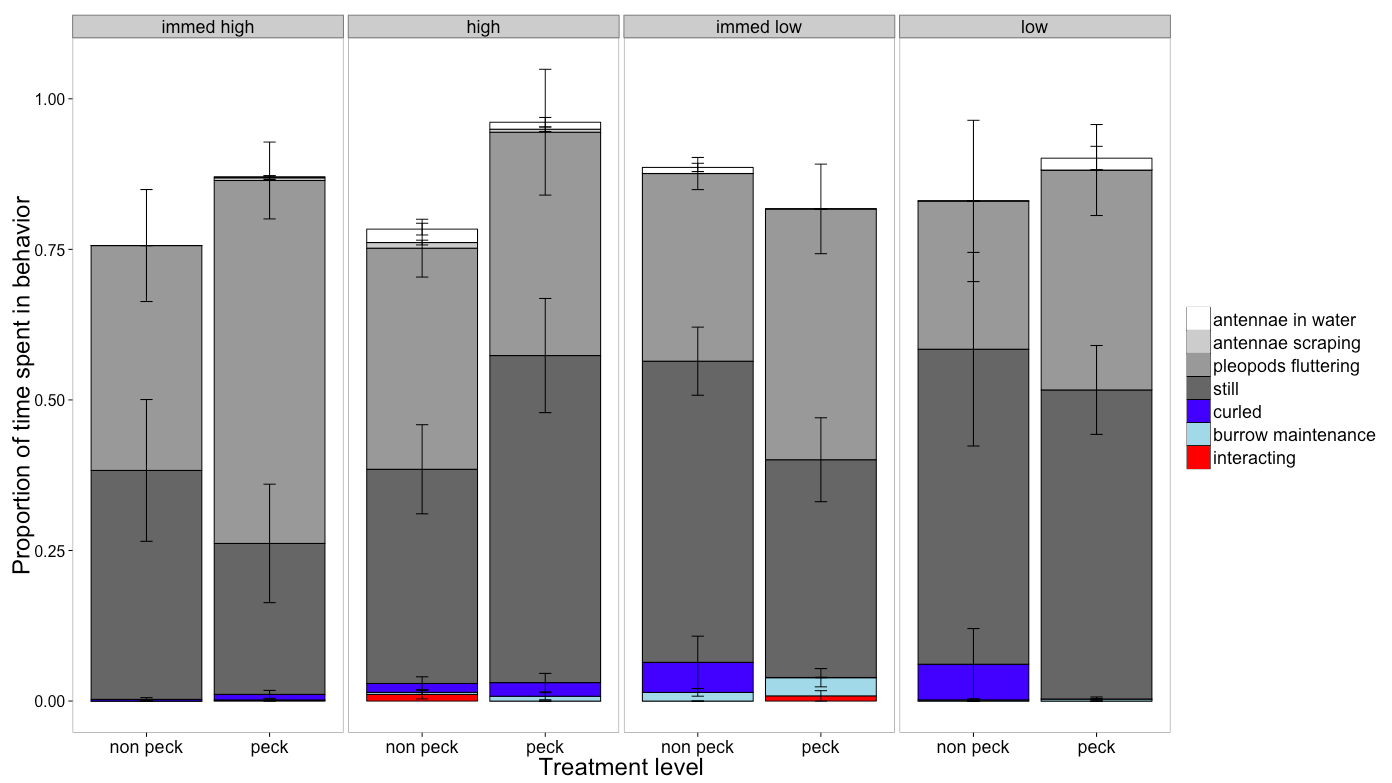


**Figure S2.1.** **Proportion of time *Corophium volutator* individuals spent in each of seven behaviors while in their burrow in the presence and absence of simulated shorebird pecking in a laboratory experiment.**

We monitored behavior over the course of a tidal cycle (20-min observation periods in each tidal stage per day conducted over 4 days; tidal stages: immediately high, high, immediately low, and low). To mimic natural conditions of shorebird predation, pecking occurred for the first 5 min of immediately low tide at a rate of one peck per s. Each section of a stack represents the mean proportion of time spent engaged in the corresponding behavior. Stacks do not sum to 1, because individuals were not always visible for behavior to be recorded. Error bars represent ± 1 SE, n = 6 experimental units.


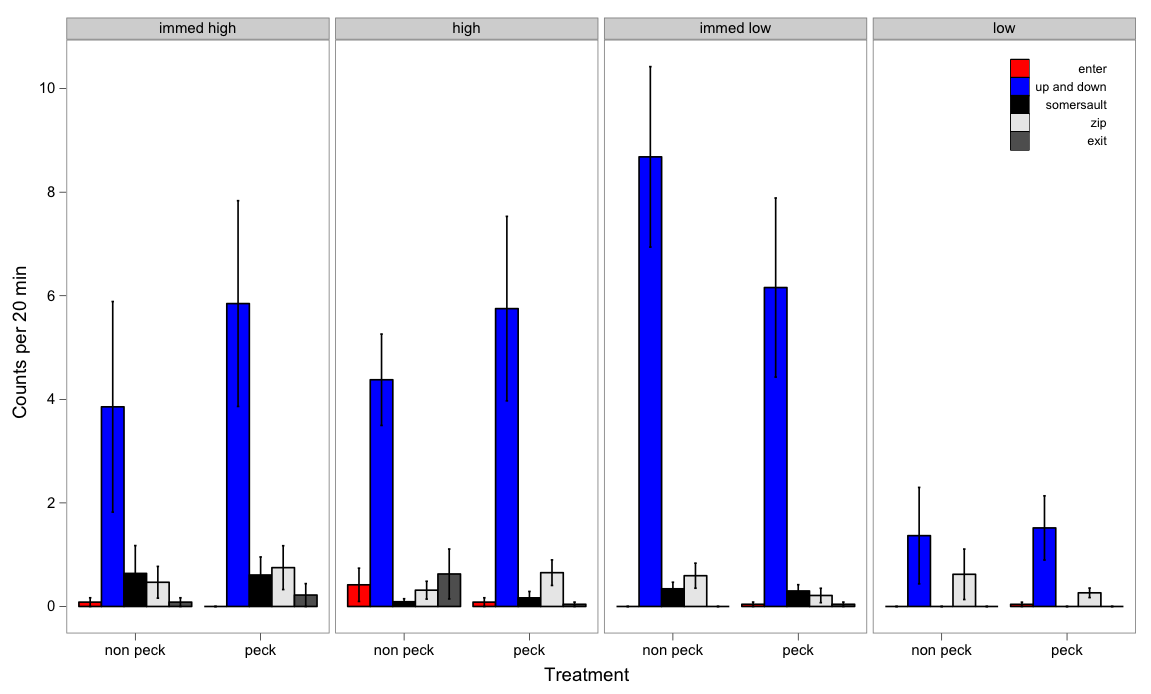


**Figure S2.2.** **Counts per 20 min of observation that *Corophium volutator* individuals spent in each of five types of movement in the presence and absence of simulated shorebird pecking in a laboratory experiment.**

We monitored behavior over the course of a tidal cycle (20-min observation periods in each tidal stage per day conducted over 4 days; tidal stages: immediately high, high, immediately low, and low). To mimic natural conditions of shorebird predation, pecking occurred for the first 5 min of immediately low tide at a rate of one peck per s. Bars represent the mean number of times *C. volutator* individuals were observed engaging in a particular movement; error bars represent ± 1 SE, n = 6 experimental units.

Reference:

1. Meadows PS, Reid A (1966) The behaviour of *Corophium volutator* (Crustacea: Amphipoda). J Zool Lond 150: 387-399.
