# Supplementary material for: Behavioral Response of Corophium volutator to Shorebird Predation in the Upper Bay of Fundy, Canada
Source: PLoS One. 2014 Oct 29;9(10):e110633. doi: 10.1371/journal.pone.0110633 (PMC4212999; doi:10.1371/journal.pone.0110633)
Supplement: Figure S3 — Proportional vertical distribution of Corophium volutator adults sampled from the predator exclusion experiment at Pecks Cove in 2007. Proportion of C. volutator sized A) 4–6 mm and B) >6 mm in bird exclosures (−B) and control plots (+B) found in each vertical layer of sediment. Layers 1–4 are 0–5, 0.5–1.5, 1.5–3.0, and 3.0–5.0 cm from the sediment surface, respectively. (DOCX) [file pone.0110633.s003.docx]

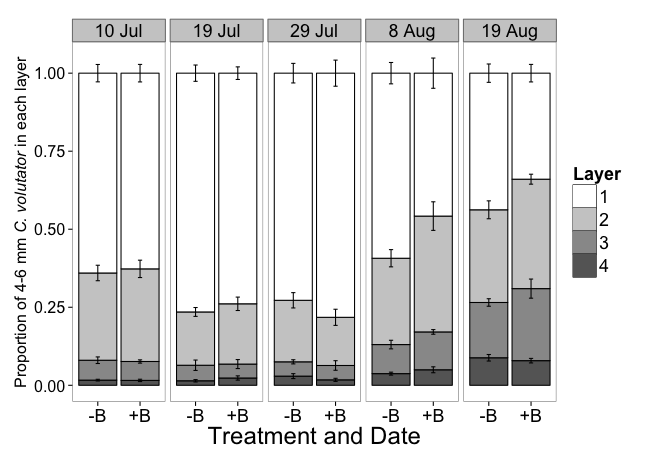


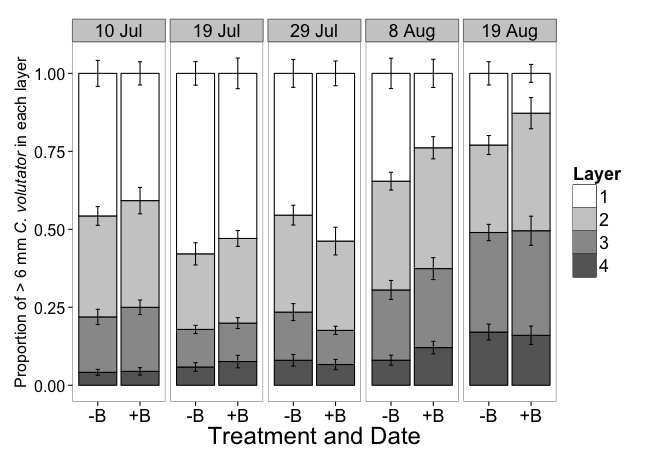


A)

B)

**Figure S3. Proportional vertical distribution of *Corophium volutator* adults sampled from the predator exclusion experiment at Pecks Cove in 2007.**

Proportion of *C. volutator* sized A) 4-6 mm and B) > 6 mm in bird exclosures (-B) and control plots (+B) found in each vertical layer of sediment. Layers 1-4 are 0-0.5, 0.5-1.5, 1.5-3.0, and 3.0-5.0 cm from the sediment surface, respectively.
