# Supplementary material for: Behavioral Response of Corophium volutator to Shorebird Predation in the Upper Bay of Fundy, Canada
Source: PLoS One. 2014 Oct 29;9(10):e110633. doi: 10.1371/journal.pone.0110633 (PMC4212999; doi:10.1371/journal.pone.0110633)
Supplement: Figure S4 — Population structure of Corophium volutator at Pecks Cove in summer 2011. Sample sizes represent the number of individuals measured (pooling over cores) to generate the size frequency distribution in each sampling round. (DOCX) [file pone.0110633.s004.docx]

**Supporting Information 4. Population structure of *Corophium volutator* in summer 2011, in the Pecks Cove mudflat, Bay of Fundy, Canada**

Methods: We assessed *Corophium volutator* population structure at Pecks Cove in 2011 to accompany casts of *C. volutator* burrows. During 3 sampling rounds (25 July, 19 August and 30 August), we made casts of *C. volutator* burrows at 3 randomly chosen locations between 100 and 300 m from shore, separated by at least 100 m (see Methods of paper for details). At the same time, using a 7-cm diameter corer, we collected 2 replicate core samples at each sampling location (totaling 6 cores per round). We washed each core through a 250-μm sieve [1], preserved the contents in 95% ethanol and counted and measured all *C. volutator* individuals (from rostrum to telson) in each sample.


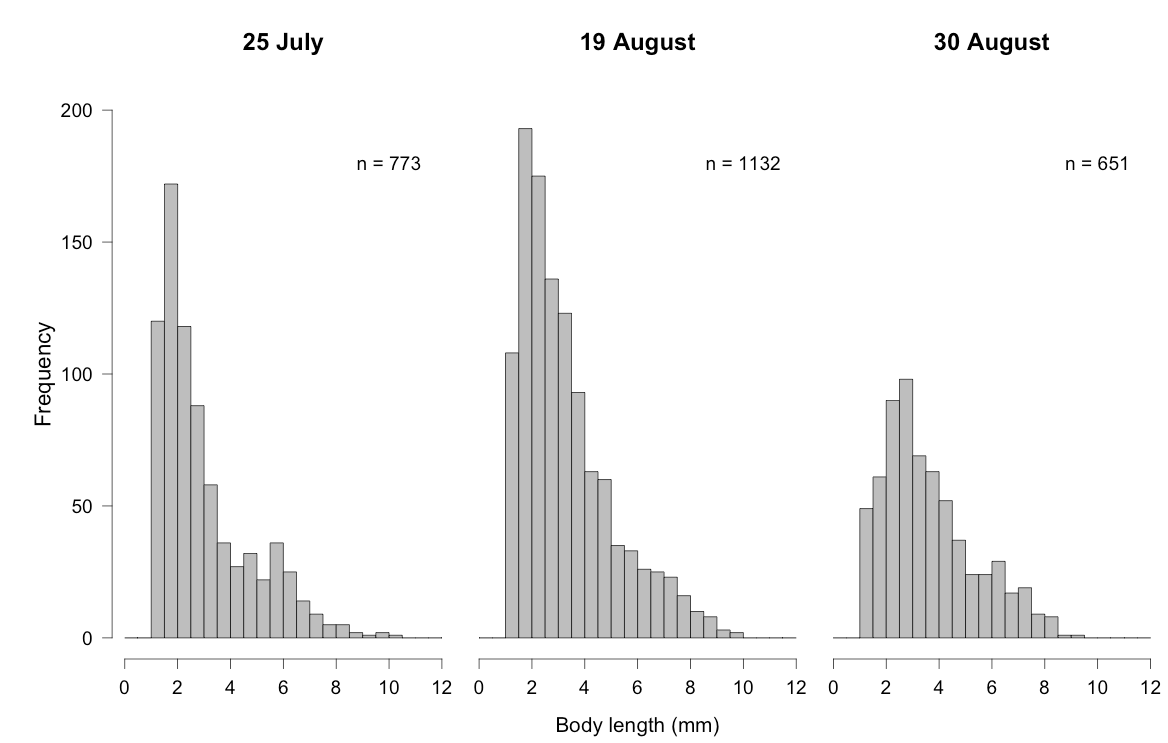


**Figure S4.** **Population structure of *Corophium volutator* at Pecks Cove in summer 2011.**

Sample sizes represent the number of individuals measured (pooling over cores) to generate the size frequency distribution in each sampling round.

Results: *C. volutator* density varied slightly among sampling rounds (mean ± SD: 25 July = 40,838 ± 10,651; 19 August = 50,583 ± 4,140; 30 August = 31,636 ± 2,127 ind./m^2^). Population structure was generally similar among rounds, other than the mode body size of juvenile amphipods increased.

Reference:

1. Crewe TL, Hamilton DJ, Diamond AW (2001) Effects of mesh size on sieved samples of *Corophium volutator*. Estuar Coast Shelf Sci 53: 151-154.
